# NFATc1 is a tumor suppressor in hepatocellular carcinoma and induces tumor cell apoptosis by activating the FasL‐mediated extrinsic signaling pathway

**DOI:** 10.1002/cam4.1716

**Published:** 2018-08-07

**Authors:** Sanrong Xu, Penghao Shu, Song Zou, Xiaofeng Shen, Yuanqian Qu, Yong Zhang, Kang Sun, Jin Zhang

**Affiliations:** ^1^ Department of General Surgery Affiliated Hospital of Jiangsu University Zhenjiang China; ^2^ Department of Hepatobiliary Surgery People's Hospital of Danyang Danyang China

**Keywords:** apoptosis, Fas ligand, hepatocellular carcinoma, nuclear factor of activated T‐cell cytoplasmic 1, tumor suppressor

## Abstract

Nuclear factor of activated T cells (NFAT) is a family of transcription factors that have important functions in many tumors. However, the expression level and functional role of NFAT in hepatocellular carcinoma (HCC) remain unclear. In this study, we showed that NFATc1 expression was decreased in both HCC tissues and cell lines. Low expression of NFATc1 was correlated with larger tumor size, advanced tumor‐node‐metastasis (TNM) stage, high serum AFP level, and liver cirrhosis. Furthermore, patients with low NFATc1 expression exhibited poor prognosis. Ectopic expression of NFATc1 in HCC cells inhibited proliferation and colony formation, leading to G1 arrest and induction of apoptosis. In addition, we demonstrated that NFATc1 increased Fas ligand (FasL) expression by directly binding to its promoter and activated the extrinsic apoptotic pathway. NFATc1 and FasL expression patterns and their prognostic value for patients with HCC were also evaluated in TCGA Liver Hepatocellular Carcinoma database. Knock‐down of FasL expression by siRNA in HCC cell lines abolished NFATc1's antiproliferative and pro‐apoptotic effects. In conclusion, NFATc1 is frequently inactivated in HCC and functions as a tumor suppressor in liver carcinogenesis. Ectopic expression of NFATc1 in HCC cells induces apoptosis by activating the FasL‐mediated extrinsic signaling pathway.

## 1 INTRODUCTION

Hepatocellular carcinoma (HCC) is one of the most common malignant tumors in the world.^1^ Despite advances in prevention strategies and in screening and technology applied during both diagnosis and treatment, HCC incidence and mortality continue to rise.^2^ Hence, there is an urgent need to clarify the complex molecular mechanisms involved in HCC carcinogenesis and progression to identify new biomarkers for early detection and targeted therapeutic drugs.

Nuclear factor of activated T cells (NFAT) was originally described as an essential transcription factor for T‐cell activation and differentiation.^3^ In humans, the NFAT family comprises five distinct gene products: nuclear factor of activated T‐cell cytoplasmic 1 (NFATc1) (also known as NFAT2 and NFATc), NFATc2 (also known as NFAT1 and NFATp), NFATc3 (also known as NFAT4 and NFATx), NFATc4 (also known as NFAT3), and NFAT5 (also known as TonEBP and OREBP). Each family member has several isoforms that arise through alternative splicing.^4^ In the basal state, NFAT is hyperphosphorylated within the cytoplasm. When cells are stimulated, NFAT is dephosphorylated by the phosphatase calcineurin and translocates to the nucleus, where it cooperates with other essential transcription factors to promote gene transcription.^5^

Many studies suggest that NFAT not only regulates many kinds of immune cells^6^, ^7^, ^8^, ^9^, ^10^ but also functions in nonimmune cells, including the bone, heart, pancreas, and skin.^11^, ^12^, ^13^, ^14^ Moreover, recent research found that NFAT regulated cell proliferation, invasion, differentiation, and angiogenesis in many types of cancer cells, including colon cancer, breast cancer, pancreatic cancer, lung cancer, ovarian cancer, and melanoma.^15^, ^16^, ^17^, ^18^, ^19^, ^20^ However, little information is available regarding NFAT expression and function in HCC. Hence, in our study, we investigated NFAT family expression in HCC and adjacent nontumor tissues and found that NFATc1 abundance was very different between tumor and nontumor tissues. We further demonstrated that NFATc1 expression was very low in both HCC tissues and cell lines. Next, we explored the relationship between NFATc1 expression and clinical parameters to evaluate the prognostic value of NFATc1 for the survival of patients with HCC. We then examined the function of NFATc1 in HCC cell lines using cell proliferation, colony formation, cell cycle, and apoptosis assays. Finally, we investigated possible underlying mechanisms for how NFATC1 induces apoptosis in HCC cells.

## 2 MATERIALS AND METHODS

### 2.1 Tissue samples and cell lines

Hepatocellular carcinoma samples, including tumor (T), adjacent nontumor (NT), and normal (N) liver tissues, were obtained from 101 patients who received hepatectomy between 2009 and 2018 at the Affiliated Hospital of Jiangsu University. In 134 cases, 81 HCC specimens, including T and NT tissues, and 20 normal liver tissues, were subjected to immunohistochemistry (IHC) to evaluate NFATc1 expression and to define clinical parameters. Thirty‐three HCC specimens, including T and NT tissues, were subjected to IHC to examine FasL expression. No chemotherapy or radiotherapy was performed before surgery. Adjacent nontumor tissues are at least 2 cm from the edge of the tumor. All tumor specimens were pathologically diagnosed as hepatocellular carcinoma. In 20 tumor‐free normal tissues, 8 cases were pathologically diagnosed as hepatic hemangioma and 12 cases as hepatolithiasis.

The patients with HCC are grouped by clinical parameters including age, sex, tumor size, tumor multiplicity, major vessel invasion, histologic grade, tumor‐node‐metastasis (TNM) stage, HBsAg status, HCVAb status, alpha‐fetoprotein (AFP), liver cirrhosis, and child classification. The number of patients in each group is presented in Table 1. TNM stage was defined according to the American Cancer Joint Committee (AJCC)'s Cancer Staging System (7th Edition). Histologic grade of tumor differentiation was assigned by the Edmondson grading system. This study was approved by the Research Ethics Committee of the Affiliated Hospital of Jiangsu University, and informed consent was obtained from each patient.

**Table 1:** Correlations between NFATC1 expression and clinical parameters

Characteristic	All cases	Protein expression		*P* value
		Low	High	
Age (y)				
≤50	40	26	14	0.193
>50	41	32	9	
Sex				
Female	22	13	9	0.127
Male	59	45	14	
Tumor size (cm)				
**≤**5	33	19	14	0.020^a^
>5	48	39	9	
Tumor multiplicity				
Single	69	48	21	0.329
Multiplicity	12	10	2	
Major vessel invasion				
No	79	56	23	0.367
Yes	2	2	0	
Histologic grade				
G1	18	12	6	0.627
G2	40	28	12	
G3	14	12	2	
G4	9	6	3	
TNM stage				
I	34	17	17	0.001^a^
II	32	28	4	
III	15	13	2	
HBsAg status				
Negative	20	14	6	0.887
Positive	60	43	17	
HCVAb status				
Negative	78	55	23	0.266
Positive	3	3	0	
AFP (ng/mL)				
≤200	51	32	19	0.021^a^
>200	30	26	4	
Liver cirrhosis				
No	31	18	13	0.033^a^
Yes	50	40	10	
Child classification				
A	49	35	14	0.793
B	24	18	6	
C	8	5	3	

AFP, alpha‐fetoprotein; HBsAg, hepatitis B virus surface antigen; HCVAb, hepatitis C virus antibody; major vessel refers to right or left portal vein.

No patients were TNM stage IV.

a *P *<* *0.05.

Four HCC cell lines, PLC (#TCHu119), HepG2 (#TCHu 72), Huh7 (#SCSP‐526), Hep3B (#TCHu106), and one normal liver cell line L02 (#GNHu 6) were purchased from the cell bank of the Chinese Academy of Sciences (Shanghai, China) for use in this study. Cell lines were maintained in minimum essential medium (MEM) (Gibco, #11095080, Grand Island, NY, USA) or Dulbecco's modified Eagle medium (DMEM) (Gibco, #10569010, USA) supplemented with 10% fetal bovine serum (Gibco, #10099141, USA).

### 2.2 RNA extraction and quantitative reverse transcription polymerase chain reaction (qRT‐PCR)

Protocols for qRT‐PCR were described previously.^21^ Total RNA was extracted with Multisource Total RNA Miniprep (Axygen, #AP‐MN‐MS‐RNA‐50, Tewksbury, MA, USA) from tissues or cell lines according to the manufacturer's instructions. Then, 1 μg total RNA was reverse‐transcribed into cDNA with PrimeScript RT reagent Kit with gDNA Eraser (Takara, #RR047A, Tokyo, Japan) according to the manufacturer's instructions. Real‐time PCR was performed using SYBR^®^ Premix Ex Taq II (Takara, #RR820L, Japan) in a MX3000P instrument (Stratagene, Santa Clara, CA, USA) according to the manufacturer's protocol. PCR primer sequences are shown in Table 2. Quantitative real‐time PCR reactions (total volume 20 μL) contained 0.5 μL of cDNA, 10 μL of SYBR^®^ Premix Ex Taq II, 0.8 μL of each 10 μmol/L forward primer and reverse primer, 0.4 μL of 50X ROX reference dye, and 8.3 μL DNase‐free water. Real‐time PCR cycles were performed as follows: 95°C for 30 seconds, 40 cycles of 95°C for 5 seconds, 60°C for 30 seconds, and 72°C for 30 seconds and a dissociation stage. β‐actin mRNA was used for normalization. Assays were repeated three times.

**Table 2:** PCR primer sequences used in this study

Target gene	Gene ID	Application	Sequence (5′‐3′)
ACTB	60	Real‐time PCR	F: GGACCTGACTGACTACCT
			R: CTTAATGTCACGCACGATT
NFATC1	4772	Real‐time PCR	F: TTCGGAATCAGAGGATAA
			R: AGGCTCATAATCATCAGT
NFATC1	4772	RT‐PCR	F: TAGCTCGAGCTATGCCAAGCACCAGCTTTCC
			R: TATGGATCCCTGCTGTGGCAGCAGGGC
MAT1A	4143	CHIP‐qPCR	F: AGACCAATCCAGATGAGAC
			R: GTAGTGCTCCAGAGTTCA
FASLG	356	CHIP‐qPCR	F: CACTGACCATTCTCCTGTAG
			R: GCTATGCTCACCTTCCTG
MAT1A	4143	Real‐time PCR	F: GAGAGTGCTTGTCCAGGTT
			R: GAGAGGTTCCGTAGGTGAAG
MAT2A	4144	Real‐time PCR	F: CAATCTACCACCTACAGCCAAGT
			R: ACCGCCATAAGTGTCCACAAT
MAT2B	27430	Real‐time PCR	F: GCAGTTCAGCAACAAGTC
			R: GATCCAGCATTCTCTTCTCT
FASLG	356	Real‐time PCR	F: GTTCTGGTTGCCTTGGTAG
			R: CATCTGGCTGGTAGACTCT
FAS	355	Real‐time PCR	F: GCCAATTCTGCCATAAGC
			R: TTGTCTGTGTACTCCTTCC
BCL2L1	598	Real‐time PCR	F: GTGCGTGGAAAGCGTAGA
			R: CTAGGTGGTCATTCAGGTAAGTG
GPC3	2719	Real‐time PCR	F: GGACAACGAGATAAGCACCTT
			R: AAGCACACCACCGAGATG
CYCS	54205	Real‐time PCR	F: GGAGGCAAGCACAAGACT
			R: TTATTGGCGGCTGTGTAAGA
BCL2	596	Real‐time PCR	F: TGTGTGGAGAGCGTCAAC
			R: CGGTTCAGGTACTCAGTCAT
CASP8	841	Real‐time PCR	F: GGAACAACTGGACAGTGAAG
			R: CCTGGAGTCTCTGGAATAACA
CASP9	842	Real‐time PCR	F: CGAACTAACAGGCAAGCA
			R: AATCCTCCAGAACCAATGTC
VEGFA	7422	Real‐time PCR	F: CTGCTCTACCTCCACCAT
			R: ACCACTTCGTGATGATTCTG
KDR	3791	Real‐time PCR	F: GGAGTCTGTGGCATCTGAAG
			R: CGGTGGTGTCTGTGTCATC
BAX	581	Real‐time PCR	F: CAAGAAGCTGAGCGAGTGT
			R: GGCGGCAATCATCCTCTG
BAK1	578	Real‐time PCR	F: CCGACGCTATGACTCAGAG
			R: TGGTGGCAATCTTGGTGAA
DNMT1	1786	Real‐time PCR	F: CGGCTTCAGCACCTCATT
			R: AGGAACTCCACCACAATCTTG
FASLG promoter	356	RT‐PCR	F: TATGGTACCCAGTTGGGCTCAGTGCAGT
			R: CCGCTCGAGCTCTCGGAGTTCTGCCAGC

### 2.3 Western blot

Total protein was extracted from tissues and cells lines using RIPA lysis buffer (Sigma, #V900854, St. Louis, MO, USA) with protease inhibitors (Sigma, #P8340, USA). Protein (40 μg) from each sample was first separated by sodium dodecyl sulfate 10% polyacrylamide gel electrophoresis (10% SDS‐PAGE). Then, a gel and membrane sandwich was made in a tank, and protein was wet‐transferred to a 0.45 μm PVDF membrane (Millipore, #IPVH00010, Billerica, MA, USA) in a transfer buffer (Beyotime, #P0021A, Shanghai, China) at constant current 300 mA for 2 hours. Membranes were incubated in blocking solution (5% milk/TBST) for 1 hour and subsequently incubated with primary antibodies diluted in 5% TBST at 4°C overnight and secondary antibodies diluted in 5% TBST at room temperature for 1 hour. Proteins were visualized using Immobilon™ Western Chemiluminescent HRP Substrate (Millipore, #WBKLS0500, USA) and recorded by FluorChem FC3 system (ProteinSimple, San Jose, CA, USA). Antibodies used in this experiment are listed in Table 3. Assays were repeated three times.

**Table 3:** Antibodies used in this study

Antibody name	Source	Dilution rate
NFATc1	Abcam (ab2796)	1:1000 for Western
FasL	Abcam (ab15285)	1:1000 for Western
β‐actin	Sigma‐Aldrich (A5316)	1:10000 for Western
Caspase‐8	Abcam (ab25901)	1:1000 for Western
Caspase‐3	Abcam (ab13847)	1:1000 for Western
Caspase‐9	Abcam (ab32539)	1:1000 for Western
HRP‐labeled goat anti‐mouse IgG (H + L)	Beyotime (A0216)	1:1000 for Western
HRP‐labeled goat anti‐rabbit IgG (H + L)	Beyotime (A0208)	1:1000 for Western

### 2.4 Immunohistochemistry (IHC)

Protocols for immunohistochemistry were described in our previous article.^21^ All of the following procedures were performed using UltraSensitive S‐P kit (MaixinBio, #KIT‐9720, Fuzhou, China) according to the manufacturer's instructions. Briefly, tissue samples were fixed by soaking in formalin overnight and embedded in paraffin. Then tissues were sectioned at 4 μm thicknesses, deparaffinized in xylene and rehydrated by graded ethanol. Tissue section was incubated with 50 μL of 0.3% hydrogen peroxide at room temperature for 10 minutes to block endogenous peroxidase activity and was boiled in EDTA (1 mmol/L, pH 6.0) in a microwave oven for 10 minutes for antigen retrieval. After rinsing with phosphate‐buffered saline (PBS) (Beyotime, #C0221A, China), slides were first incubated with goat normal nonimmune serum for 15 minutes. Subsequently, slides were incubated with mouse anti‐NFATc1 antibody (Abcam, #ab2796, Cambridge, UK) diluted in PBS (1:100) or rabbit anti‐FasL antibody (Abcam, # ab15285, UK) diluted in PBS (1:100) at 4°C overnight. Then, slides were incubated with a biotinylated secondary antibody at 37°C for 30 minutes and then incubated with a streptavidin‐horseradish peroxidase tertiary antibody at 37°C for 30 minutes. Finally, protein color staining was visualized using DAB Horseradish Peroxidase Color Development Kit (Beyotime, #P0203, China). All slides were counterstained in hematoxylin, dehydrated, and covered with neutral balsam. Antibodies used in this experiment are listed in Table 3.

### 2.5 IHC evaluation

Immunostaining scores were assigned according to the positive percentage and intensity of staining.^22^ The positive percentage was scored as “0” (0%), “1” (0%–10%), “2” (10%–50%), or “3” (50%–100%). Staining intensity was scored as “0” (negative), “1” (weak), “2” (moderate), and “3” (strong). The two scores were multiplied, resulting in final scores ranging from 0 to 9. Immunostaining results were scored by two experienced pathologists who were blinded to clinical data. For statistical analysis, scores of 0‐4 were considered low expression, while scores of 5‐9 were considered high expression.

### 2.6 NFATc1 expression plasmid construction and transfection

Total RNA extracted from Huh7 cells was transcribed into cDNA using the PrimeScript™ II 1st Strand cDNA Synthesis Kit (Takara, # 6210A, Japan). DNA corresponding to the coding sequence (CDS) of NFATc1 isoform B was obtained by RT‐PCR amplification of Huh7 cDNA using PrimeSTAR^®^ Max DNA Polymerase Kit (Takara, # R045A, Japan). Primers used in the experiment are listed in Table 2. PCR products were digested with Xho I (Takara, #1635, Japan) and BamH I (Takara, #1605, Japan) and ligated with the mammalian expression vector pcDNA3.1B‐FLAG‐GFP (pcDNA3.1) using DNA Ligation Kit (Takara, #6023, Japan). This vector derived from pcDNA3.1/Myc‐HisB (Invitrogen, # V85520, Carlsbad, CA, USA) was a kind gift from Dr B‐F Wei (National Human Genome Center, Shanghai). All constructed plasmids were verified by DNA sequencing. For plasmid transfection, expression plasmids pcDNA3.1‐NFATc1 (NFATc1) or empty vector pcDNA3.1 for negative controls (NC) were transfected into HCC cells. Transfection was performed using the ViaFect reagent (Promega, #E4982, Madison, WI, USA) according to the manufacturer's instructions. Effective FasL siRNA (5′‐GGAAGACACCUATGGAAUU‐3′) (si‐FasL) and negative control siRNA(5′‐GGCUCUAGAAAAGCCUAUGC‐3′) (si‐NC) were designed and synthesized by Ribobio Co., Ltd. (Guangzhou, China). siRNA was transfected using the ribo FECT ™ CP Transfection Kit (Ribobio, #C10511‐1, China) according to the manufacturer's instructions.

### 2.7 Colony formation assay

Colony formation assays were performed using monolayer culture. Cells (2 × 10^5^/well) were plated in 6‐well plates and transfected with 2 μg expression plasmids pcDNA3.1‐NFATc1 (NFATc1) or empty vector pcDNA3.1 for negative controls (NC) using ViaFect (Promega, #E4982, USA). Forty‐eight hours after transfection, NFATc1 and NC groups were determined to have the same transfection efficiency by detecting GFP in a fluorescence microscope. Cells were then refreshed with culture media, and transfected cells were selected using 500 μg/mL G418 for 10 days. Finally, more than 50 cells were considered as a colony, and colony number was counted after staining with Giemsa solution (Sigma, #G5637, USA). All experiments were performed in triplicate wells, and assays were repeated three times.

### 2.8 Cell proliferation assay

Cell proliferation was measured by the CCK‐8 assay (Dojindo, #CK04, Kumamoto, Japan) according to the manufacturer's instructions. Briefly, cells (1 × 10^3^/well) were seeded into 96‐well plates and transfected with expression plasmids pcDNA3.1‐NFATc1 or empty vector pcDNA3.1. In the following 1, 2, 3, and 4 days after transfection, 90 μL of complete culture medium plus 10 μL of CCK‐8 solution was added to 96‐well plates. After incubation at 37°C for 1 hour, sample absorbance was measured at 450 nm on a microplate reader (Multiskan FC, Thermo Fisher Scientific Waltham, MA, USA). Assays were repeated three times.

### 2.9 Cell cycle detection by flow cytometry

Protocols for cell cycle detection by flow cytometry were described previously.^23^ Cells were transfected with expression plasmids pcDNA3.1‐NFATc1 or empty vector pcDNA3.1 and cultured for 2 days. Cells were then collected, washed twice with ice‐cold PBS, fixed with 70% ice‐cold ethanol, and stained with propidium iodide (PI, 50 μg/mL) (Sigma, # P4170, USA) in the presence of RNase (100 μg/mL) (Takara, # 2158, Japan). 1 × 10^5^ cells were analyzed using FACSCanto Cytometer (BD Bioscience, San Jose, CA,USA), and cell cycle profiles were analyzed by ModFit software. Assays were repeated three times. Gate strategy of cell cycle analysis by flow cytometry is presented in Figure S2.

### 2.10 Apoptosis detection by flow cytometry

Apoptosis assays were performed using the Annexin V Apoptosis Detection Kit APC (eBioscience, #88‐8007‐74, San Diego, CA, USA) according to the manufacturer's instructions. Cells were transfected with expression plasmids pcDNA3.1‐NFATc1 or empty vector pcDNA3.1. After 2 days of cell culture, cells were collected for apoptosis analysis. Briefly, cells were washed with PBS, digested with trypsin, and centrifuged. Cells were washed with PBS and binding buffer. Then, cells were suspended in binding buffer at 1‐5 × 10^6^/mL, and 5 μL of Annexin V‐APC was added to the 100 μL cell suspension. After 15 minutes incubation at room temperature, cells were centrifuged, washed with binding buffer, and suspended in 200 μL binding buffer, and 5 μL of propidium iodide staining solution was added into cell suspension. Cell suspensions were transferred into flow tubes for flow cytometry using FACSCanto Cytometer (BD Bioscience, USA). Apoptosis profiles were analyzed by FlowJo software. Assays were repeated three times. Gate strategy of apoptosis analysis by flow cytometry is presented in Figure S1.

### 2.11 FasL promoter construction and dual‐luciferase reporter assay

The FasL promoter (−1000/+542) was amplified from human genomic DNA by PCR and then inserted into the pGL3‐Basic vector to generate a FasL promoter luciferase reporter plasmid. Primers used in this experiment are listed in Table 2. To analyze FasL promoter activity, cells were seeded at a density of 1 × 10^4^ cells/well in 24‐well culture dishes. Cells were cotransfected with FasL promoter luciferase reporter plasmid and NFATc1 expression plasmids pcDNA3.1‐NFATc1 or empty vector pcDNA3.1 and Renilla luciferase reporter plasmids. Forty‐eight hours after transfection, cells were collected, and promoter activity was measured using the Dual‐Luciferase Reporter Assay kit (Promega, #E1910, USA) according to the manufacturer's protocol. Assays were repeated three times.

### 2.12 Chromatin immunoprecipitation quantitative polymerase chain reaction (ChIP‐qPCR) assay

Chromatin immunoprecipitation quantitative polymerase chain reaction assays were performed as previously described^24^, ^25^ using the EZ‐ChIP™ kit (Millipore, #17‐371, USA) according to the manufacturer's protocol. Briefly, Huh7 cells were transfected with expression plasmids pcDNA3.1‐NFATc1 or empty vector pcDNA3.1 in a 10‐cm dish. Forty‐eight hours post‐transfection, formaldehyde was added to the culture media at a final concentration of 1% and incubated at 37°C for 10 minutes. Cells were broken with SDS Lysis Buffer containing 1X Protease Inhibitor Cocktail II and sonicated on wet ice to obtain 200‐1000 bp of DNA fragment. One‐tenth of the lysate was kept to quantify the amount of DNA present in different samples before immunoprecipitation (input). Chromatin was immunoprecipitated using either NFATc1 antibody (Abcam, #ab2796, UK) (1:1000) or Normal Mouse IgG (kit provided, 1:1000) as a negative control. 8 μL 5 mol/L NaCl (final concentration of 0.2 mol/L) was added to the sample and incubated at 65°C overnight to reverse the DNA‐Protein crosslinks. Finally, immunoprecipitated DNA fragments were purified using AxyPrep PCR Clean kit (Axygen, #AP‐PCR‐50, USA) and detected by real‐time quantitative PCR assays with annealing temperature at 60°C using specific primers, which are listed in Table 2. A Ct value was calculated for each sample: ΔCt = Ct (sample)−Ct (Input). Next, a ΔΔCt value was calculated: ΔΔCt = ΔCt (sample immunoprecipitated with NFATc1 antibody)−ΔCt (sample immunoprecipitated with IgG). Fold enrichment was thus calculated as 2^−∆∆Ct^. All assays were repeated three times.

### 2.13 TCGA dataset analysis

TCGA‐HCC data including tumors and corresponding adjacent normal tissues were downloaded from the MethHc database (http://methhc.mbc.nctu.edu.tw/php/index.php). The expression pattern of NFATc1 and FasL in 50 pairs of tumors and corresponding adjacent normal tissues and correlation of their expression in 205 HCC tissues were analyzed. Kaplan‐Meier curves were used to analyze overall survival of patients, which was stratified by NFATc1 or FasL expression using the online tool (http://kmplot.com/analysis/), and the auto select best cutoff was chosen.

### 2.14 Statistical analysis

Statistical analysis was performed using SPSS 24.0 for Windows (SPSS) and GraphPad Prism 6. Results are expressed as the mean ± standard deviation (SD). For qRT‐PCR and dual‐luciferase reporter assay, quantitative data between groups were compared using the Wilcoxon matched‐pairs signed‐rank test or Mann‐Whitney *U* test according to whether or not data were paired. Other quantitative data analysis was performed using two‐tailed Student t tests. Correlation was analyzed using Spearman's rank correlation test. Overall survival curves were performed using the Kaplan‐Meier method and analyzed by the log‐rank test. *P* values of <0.05 were considered statistically significant.

## 3 RESULTS

### 3.1 NFATc1 expression is significantly low in HCC tissues and cell lines and its low expression correlates with poor survival in patients with HCC

**Figure 1:**
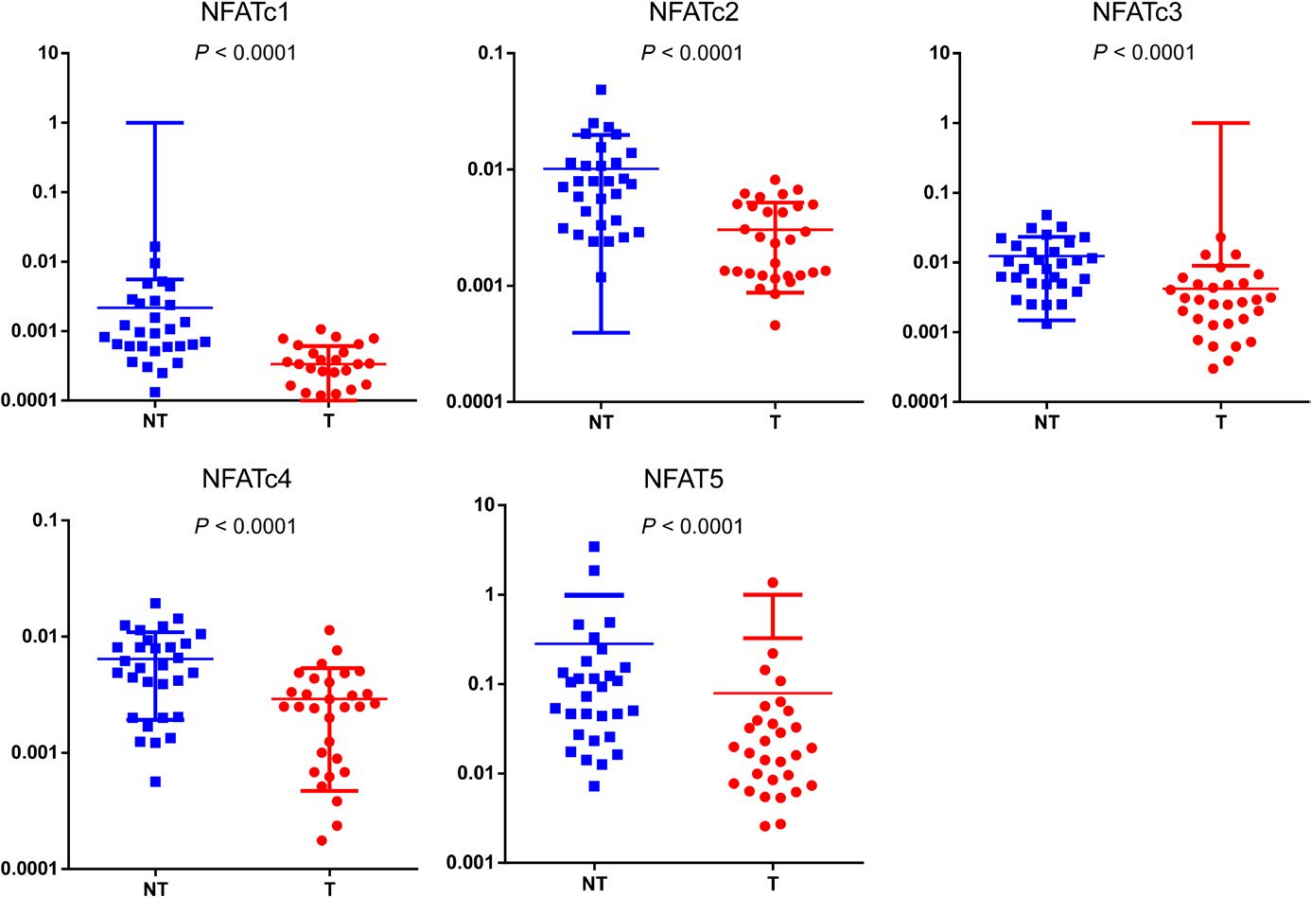
NFAT family member mRNA levels are downregulated in HCC. Expression of NFAT family members NFATc1, NFATc2, NFATc3, NFATc4, and NFAT5 mRNA in 30 pairs of HCC and corresponding adjacent nontumor liver tissues were evaluated by real‐time quantitative PCR (qPCR). NFAT family member expression was significantly downregulated in tumor tissues compared to nontumor tissues. Data are presented as NFAT expression normalized to β‐actin. T: HCC tumor tissue. NT: adjacent nontumor liver tissues. Statistical analysis was performed using Wilcoxon matched‐pairs signed‐rank test. Data are presented as the mean ± SD. Dots represent mRNA level from 30 tissues

**Figure 2:**
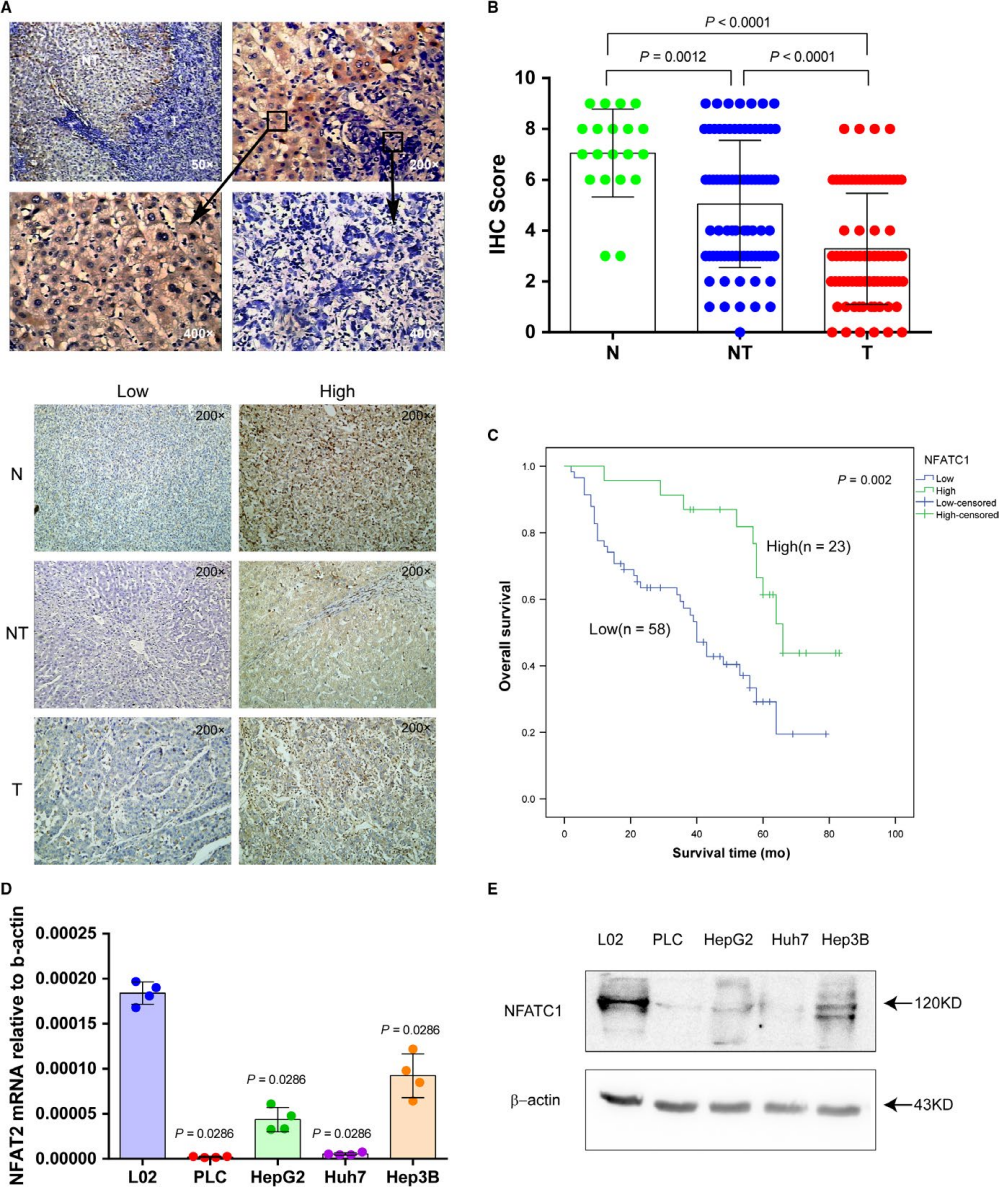
NFATc1 expression in HCC tissues and cell lines and its effect on patients with HCC prognosis. A, Representative images of NFATc1 protein expression in HCC tissues, adjacent nontumor tissues and normal liver tissues determined by immunohistochemistry (IHC). T: HCC tumor tissue. NT: adjacent nontumor liver tissues. N: normal liver tissues. B, IHC scores in HCC, adjacent nontumor liver tissues and normal liver tissues. For NT vs T, statistical analyses were performed using Wilcoxon matched‐pairs signed‐rank test. For N vs NT or T, statistical analysis was performed using the Mann‐Whitney *U* test. Dots represent IHC score from 20 normal tissues and 80 pairs of HCC and adjacent nontumor tissues. C, The prognostic value of NFATc1 expression on patient survival was calculated by the Kaplan‐Meier method and log‐rank tests. D, Relative NFATc1 mRNA expression in one normal cell line (L02) and four HCC cell lines (PLC, HepG2, Huh7, and Hep3B). Statistical analysis for L02 vs PLC, HepG2, Huh7, and Hep3B was performed by the Mann‐Whitney *U* test. β‐actin was used as an internal control. Data are presented as the mean ± SD. Dots represent data from four replicates of pipetting for measurement of qPCR. E, NFATc1 protein expression in one normal cell line (L02) and four HCC cell lines (PLC, HepG2, Huh7, and Hep3B)

We first examined messenger RNA (mRNA) expression of NFAT family members (NFATc1, NFATc2, NFATc3, NFATc4, and NFAT5) in 30 pairs of HCC tumor tissues (T) and corresponding adjacent nontumor tissues (NT) by qRT‐PCR. NFATc1, NFATc2, NFATc3, NFATc4, and NFAT5 mRNA in HCC were downregulated by 6.47‐, 3.34‐, 2.95‐, 2.21‐, and 3.57‐fold, respectively, compared to adjacent nontumor tissues. Among NFAT family members, NFATc1 mRNA exhibited the largest difference between T and NT tissues (*P *< 0.01) (Figure 1). Therefore, we focused on NFATc1 expression and function in HCC in subsequent experiments. We analyzed NFATc1 protein expression levels in 81 pairs of HCC tumor (T) tissues and corresponding adjacent nontumor (NT) tissues, as well as 20 normal (N) liver tissues, by immunohistochemistry (IHC). Consistent with our mRNA results, NFATc1 protein expression in HCC was significantly downregulated compared to corresponding adjacent nontumor tissues and normal liver tissue (Figure 2A,B). HCC patients with low NFATc1 expression (n = 58) exhibited shorter overall survival than patients in the high expression group (n = 23) (Figure 2C). Furthermore, HCC cell lines PLC, HepG2, Huh7, Hep3B had significantly downregulated NFATc1 mRNA and protein levels compared to the normal liver cell line L02 (Figures 2D,E and S3). These results suggest that aberrant gene silencing of NFATc1 occurs in HCC.

### 3.2 Low NFATc1 expression correlates with poor clinical parameters

We next explored the association of NFATc1 expression with clinical parameters in patients with HCC (Table 1). Our results demonstrated that low expression of NFATc1 was correlated with larger tumor size (*P *= 0.020), advanced TNM stage (*P *=* *0.001), higher serum AFP levels (*P *=* *0.021), and liver cirrhosis (*P *=* *0.033). There was no statistically significant difference in NFATc1 expression by age, sex, tumor multiplicity, major vessel invasion, histologic grade, HBsAg status, HCVAb status, or child classification.

### 3.3 Ectopic expression of NFATc1 inhibits HCC cell growth and induces apoptosis

**Figure 3:**
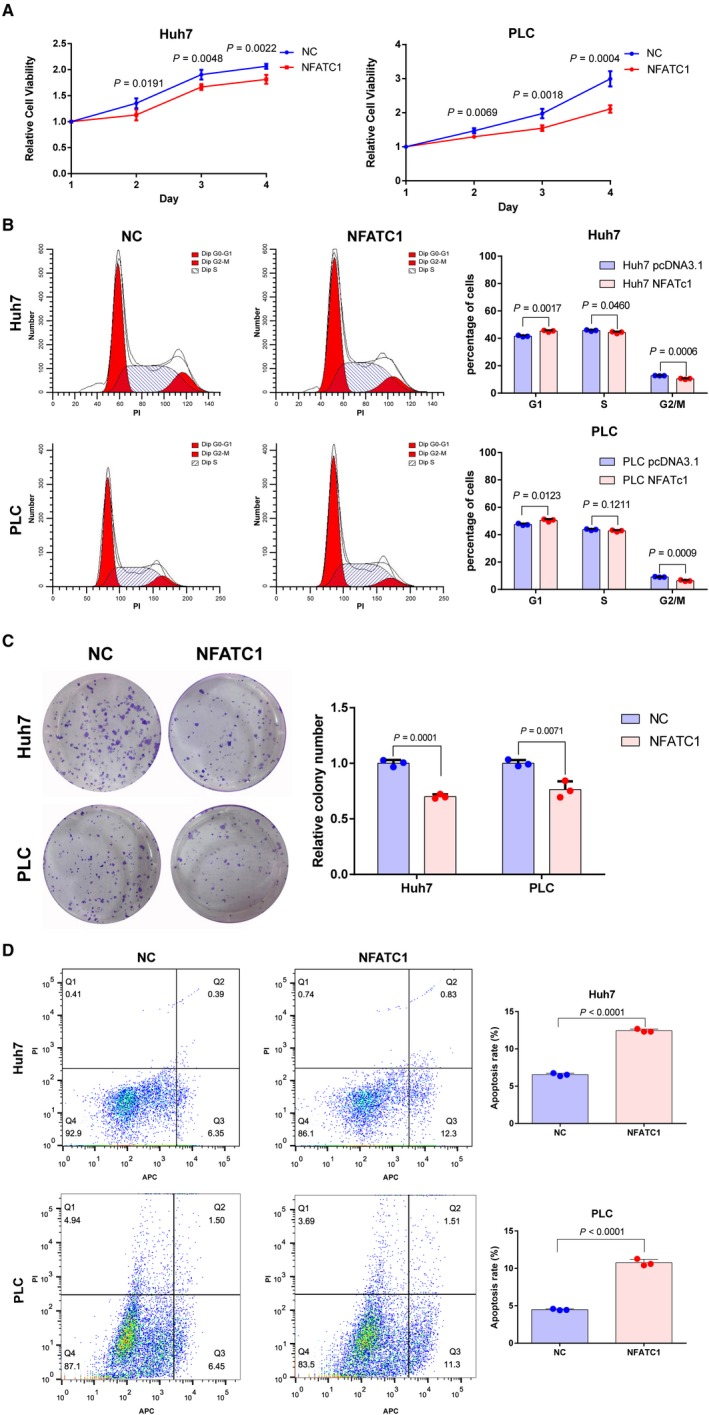
NFATc1 inhibits HCC cell proliferation and induces apoptosis. Huh7 and PLC HCC cell lines were transfected with NFATc1 overexpression plasmid (NFATc1) or vector plasmid for negative control (NC). A, Ectopic expression of NFATc1 inhibited proliferation of Huh7 and PLC cells, as shown by CCK‐8 assay. The experiments were performed in quadruplicate wells three times. Data are presented as the mean ± SD. B, Ectopic expression of NFATc1 caused G1 arrest in Huh7 and PLC cells. Images of flow cytometry analysis are shown on the left, and the mean ± SD of cell percentage for each group is shown on the right. The experiments were performed in triplicate wells three times. Data are presented as the mean ± SD. Dots represent data from cells in triplicate wells under the same treatment. C, NFATc1 inhibited colony formation in Huh7 and PLC cells. Images are shown on the left, and the mean ± SD of relative colony number for each group is shown on the right. The experiments were performed in triplicate wells three times. Data are presented as the mean ± SD. Dots represent data from cells in triplicate wells under the same treatment. D, NFATc1 induced apoptosis in Huh7 and PLC cells as shown by flow cytometry following Annexin V‐APC and PI staining. The experiments were performed in triplicate wells three times. Data are presented as the mean ± SD. Dots represent data from cells in triplicate wells under the same treatment. Statistical analysis for all the experiment data in Figure 3 was performed using two‐tailed Student *t* tests

To understand the function of NFATc1 in HCC cells, Huh7 and PLC cells with the lowest NFATc1 expression were transfected with pcDNA3.1‐NFATc1 plasmid (NFATc1) or empty vector pcDNA3.1 plasmid for negative control (NC), and CCK‐8 assays were used to evaluate proliferation levels. Our results showed that the proliferation rate of cells expressing NFATc1 was significantly lower than that of control cells at 2, 3, and 4 days post‐transfection (Figure 3A). Next, we examined the possible effects of NFATc1 on the cell cycle by flow cytometry. We found that ectopic expression of NFATc1 in HCC cell lines induced G1 phase arrest (Figure 3B). Moreover, colony formation assay results revealed that ectopic expression of NFATc1 significantly suppressed the number of cell colonies compared to the control group (Figure 3C), indicating that NFATc1 indeed suppresses proliferation of HCC cells. Furthermore, to evaluate the contribution of apoptosis to the observed growth inhibition in HCC cells mediated by NFATc1, the presence of apoptosis was examined by flow cytometry 2 days after transfection. Apoptotic cells were determined by detecting cells in the lower right (LR) and upper right (UR) quadrants of the graphs (Figure 3D), which are regarded as early‐stage and late‐stage apoptotic cells, respectively. We found the apoptosis rate of NFATc1‐expressing HCC cell lines was higher than that of control cells. These results indicate that NFATc1 inhibits HCC cell proliferation and induces apoptosis.

### 3.4 NFATc1 induces apoptosis in HCC cells by activating the FasL‐mediated extrinsic signaling pathway

**Figure 4:**
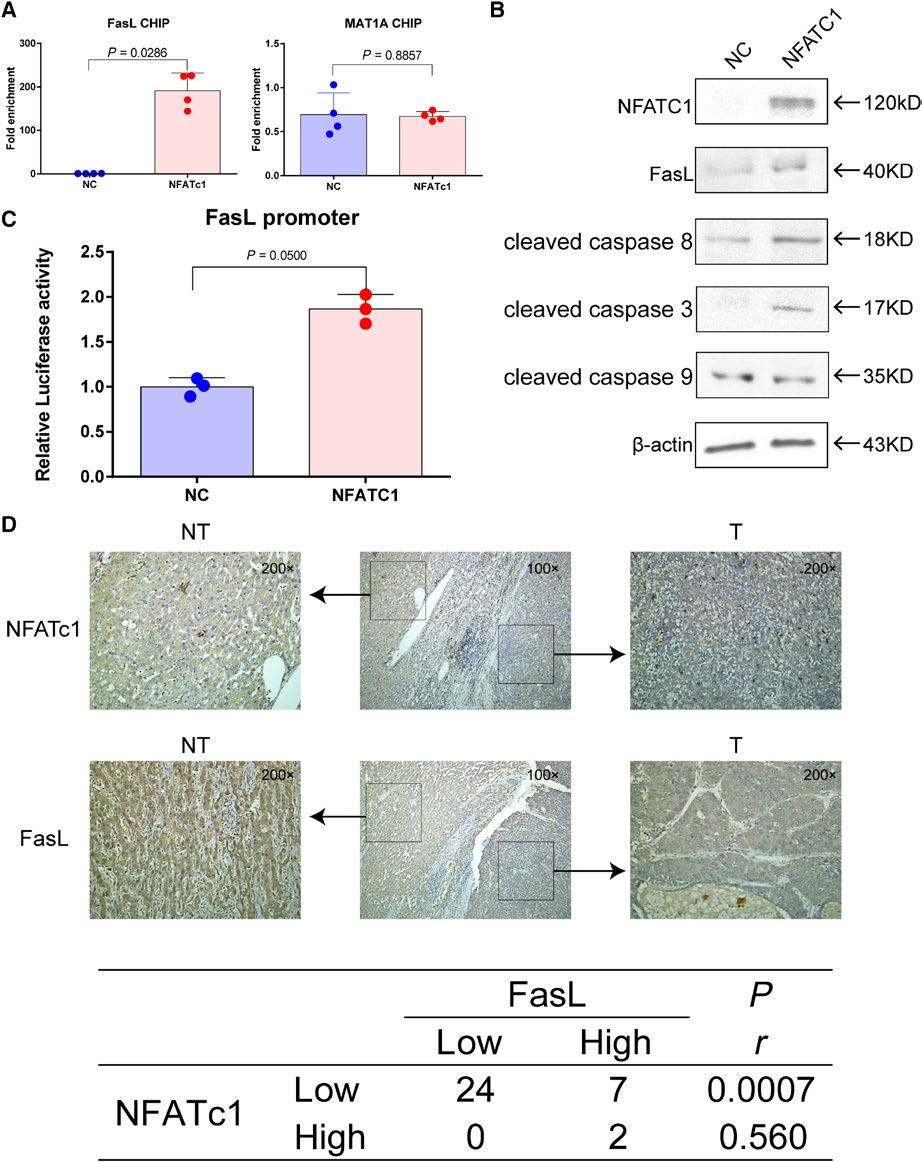
NFATc1 induces FasL expression by direct binding to the promoter and activates the extrinsic apoptotic pathway. Huh7 cells were transfected with NFATc1 overexpression plasmid (NFATc1) or vector plasmid for negative control (NC). A, NFATc1‐binding capacity to FasL and MAT1A promoters in Huh7 cells was analyzed by CHIP assay. Dots represent data from quadruplicate of pipetting for measurement of qPCR. Statistical analysis was performed using Mann‐Whitney *U* test. B, NFATc1, FasL, cleaved‐caspase 8, cleaved‐caspase 3 protein, and cleaved‐caspase 9 protein expressions were analyzed by Western blot. C, FasL promoter activity was examined by dual‐luciferase assay. Statistical analyses were performed using the Mann‐Whitney *U* test. Experiments were performed in triplicate wells three times. Dots represent data from cells in triplicate wells under the same treatment. D, Representative IHC image of NFATc1 and FasL expression in HCC and adjacent nontumor tissues from one patient. Correlation was analyzed by Spearman's rank correlation test in thirty‐three HCC tissues

To elucidate how NFATc1 inhibits HCC cell proliferation and induces apoptosis, we performed qRT‐PCR to examine possible downstream alterations in gene expression induced by ectopic expression of NFATc1. We found that NFATc1 increased the expression of both the pro‐apoptotic gene FasL and the antiproliferative gene MAT1A (Table 4). We further evaluated whether observed NFATc1‐induced FasL and MAT1A expression were associated with NFATc1 direct promoter binding. ChIP‐qPCR was performed, and our results showed that NFATc1 significantly pulled down the FasL promoter, while not exhibiting significant binding capacity for the MAT1A promoter (Figure 4A). Moreover, we used Western blot and a dual‐luciferase reporter assay to analyze FasL protein expression and promoter activity induced by NFATc1 and found that both protein expression and promoter activity were elevated after increasing NFATc1 expression in Huh7 cells (Figures 4B,C and S4). Moreover, IHC for HCC consecutive sections revealed that low NFATc1 expression was correlated with low FasL expression (Figure 4D), suggesting there is a close relationship between NFATc1 and FasL in HCC. FasL is a known key protein for triggering the extrinsic apoptosis pathway. To determine whether NFATc1 induces HCC cell apoptosis by activating the extrinsic apoptosis pathway, we examined apoptosis signaling caspase proteins (caspase 8, caspase 3, and caspase 9) by Western blot and found that ectopic expression of NFATc1 elevated expression of the active form of caspase 8 and caspase 3, but not caspase 9 (Figure 4B and S4), indicating NFATc1 induces HCC cell apoptosis by activating the FasL‐mediated extrinsic signaling pathway.

**Table 4:** Changes in gene expression induced by ectopic expression of NFATc1

Gene name	Gene ID	Function	Fold change (NFATc1/NC)	*P* value	Result
NFATc1	4772	‐	2040.91	0.002	UP
MAT1A	4143	Proliferation	1.454	0.012	UP
FASLG	356	Apoptosis	3.758	0.012	UP
FAS	355	Apoptosis	0.99	0.952	ns
BCL2L1	598	Apoptosis	1.137	0.485	ns
BCL2	596	Apoptosis	1.932	0.287	ns
Casp8	841	Apoptosis	1.212	0.252	ns
Casp9	842	Apoptosis	1.208	0.282	ns
BAX	581	Apoptosis	1.288	0.111	ns
MAT2A	4144	Proliferation	0.901	0.092	ns
MAT2B	27430	Proliferation	1.066	0.383	ns
GPC3	2719	Proliferation	0.936	0.153	ns
CYCS	54205	Apoptosis	1.253	0.145	ns
VEGFA	7422	Angiogenesis	1.037	0.716	ns
KDR	3791	Angiogenesis	0.862	0.635	ns
BAK1	578	Apoptosis	1.272	0.063	ns
DNMT1	1786	Proliferation and apoptosis	1.242	0.063	ns

Up, upregulated; ns, not significant.

### 3.5 Analysis of NFATc1 and FasL expression patterns and relationship in TCGA‐HCC database

**Figure 5:**
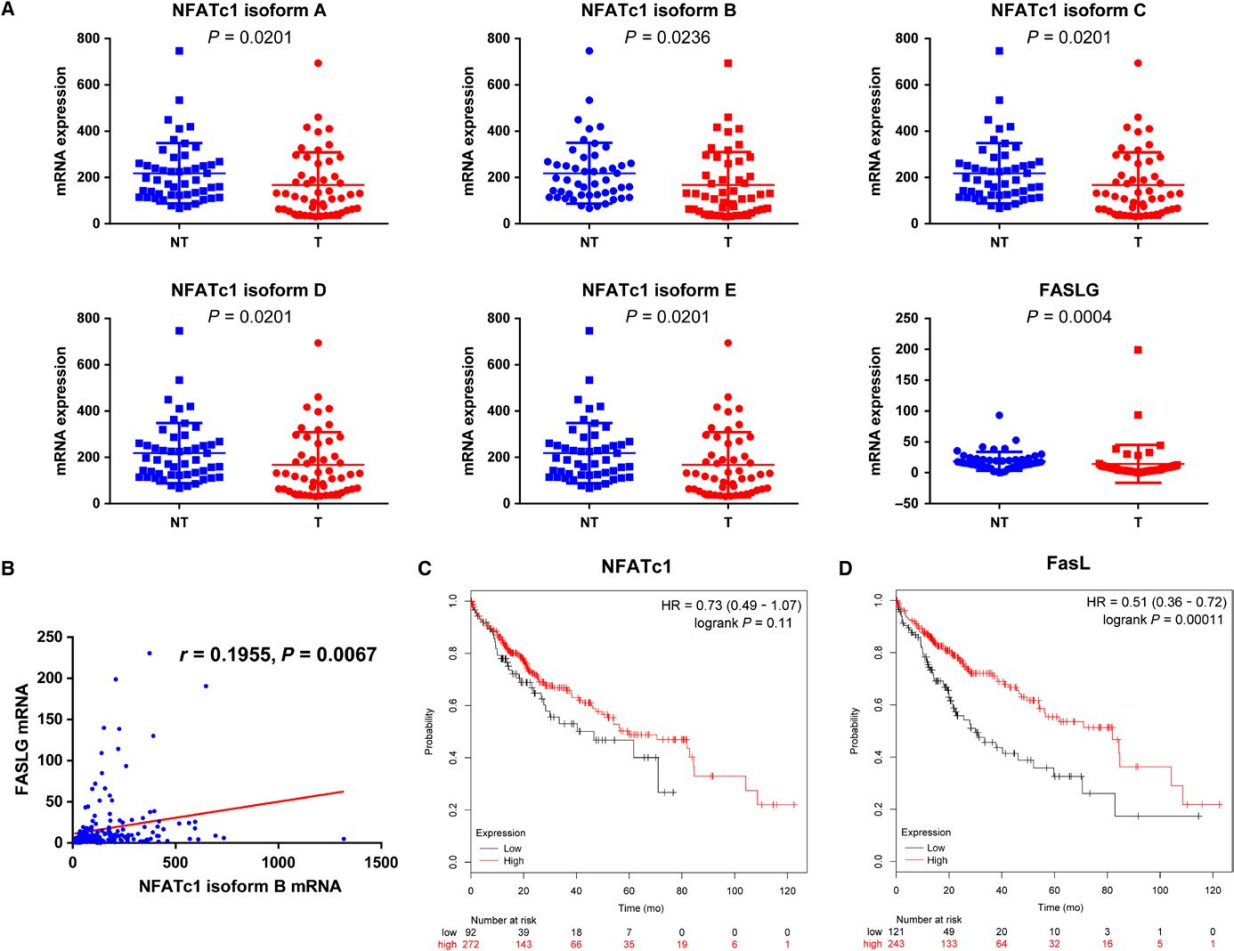
NFATc1 and FasL expression pattern and correlation in patients from TCGA‐LIHC and prognostic value for patient survival. A, NFATc1 isoforms and FasL mRNA expression in tumor and corresponding adjacent nontumor tissues from fifty patients of TCGA‐LIHC. Dots represent mRNA level from 50 pairs of HCC and adjacent nontumor tissues. Statistical analysis was performed using the Wilcoxon matched‐pairs signed‐rank test. Data are presented as the mean ± SD. B, Correlation between NFATc1 isoform B and FasL expression was analyzed by Spearman's rank correlation test (n = 205). C, D, Prognostic value of NFATc1 and FasL expression on patient survival, respectively. Statistical analysis was performed using log‐rank test

To further verify NFATc1 and FasL expression and their relationship, we accessed TCGA‐HCC database and downloaded the dataset from the MethHc database (http://methhc.mbc.nctu.edu.tw/php/index.php). These results showed that NFATc1 isoforms A, B, C, D, E and FasL mRNA were downregulated in 50 pairs of HCC tissues compared to their corresponding adjacent normal liver tissues (Figure 5A). We also found the NFATc1 isoform B mRNA was positively correlated with FasL (*r* = 0.1955, *P* = 0.0067) (Figure 5B). Further to explore the prognostic value of NFATc1 and FasL in patients with HCC, we performed Kaplan‐Meier survival analysis of based on NFATc1 or FasL expression using an online platform (http://kmplot.com/analysis/), and the autoselect best cutoff was chosen. These results showed that low NFATc1 and FasL expression were associated with worse survival in patients with HCC (*P* = 0.11, *P *=* *0.00011, respectively) (Figure 5C and 5D).

### 3.6 FasL is essential for inhibition of HCC cell growth and apoptosis induced by NFATc1

**Figure 6:**
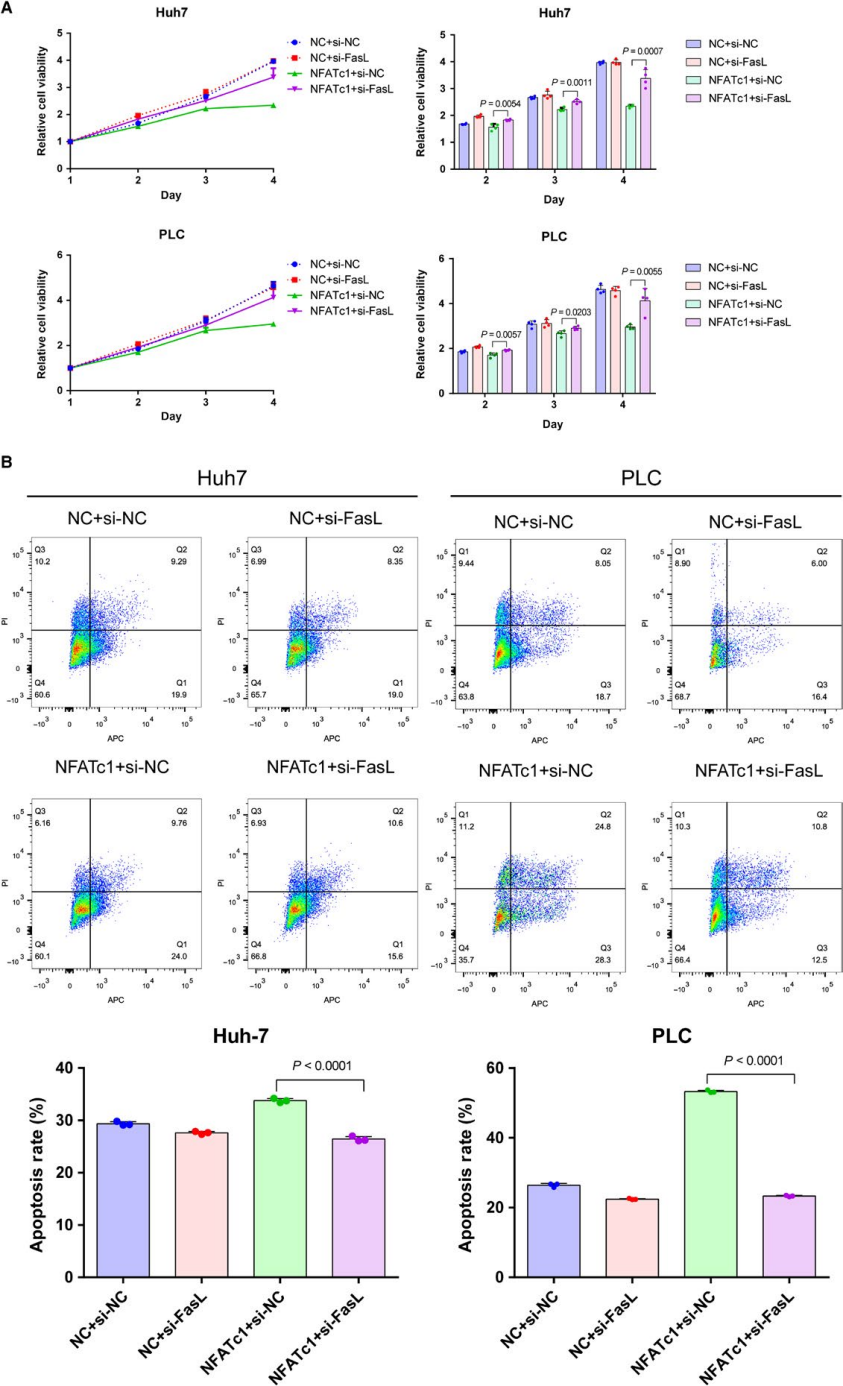
FasL is essential for HCC cell growth suppression and apoptosis induction by NFATc1. NFATc1 overexpression plasmid and FasL siRNA were cotransfected into Huh7 and PLC cells. A, Relative OD values were examined by CCK‐8 assay at various time points (1, 2, 3, and 4 d) after transfection. OD value at day 1 was used for normalization. Dots represent data from cells in quadruplicate wells under the same treatment. B, Apoptosis rates were assessed by flow cytometry at 48 h post‐transfection. Dots represent data from cells in triplicate wells under the same treatment. Statistical analysis for all the experiment data in Figure 6 was performed using two‐tailed Student *t* tests. Data are presented as the mean ± SD. All experiments were performed in triplicate wells three times

We first confirmed that the FasL siRNA can effectively knock‐down FasL mRNA in Huh7 and PLC (Figure S5). Then, to verify the role of FasL in NFATc1‐induced HCC cell growth suppression and apoptosis induction, we cotransfected NFATc1 and FasL siRNA. In the CCK‐8 assay, the results showed that knock‐down of FasL expression by siRNA significantly reduced the antiproliferative effect mediated by NFATc1 overexpression (Figure 6A). Moreover, apoptosis induced by NFATc1 overexpression was also hampered by silencing of FasL (Figure 6B). Taken together, these results confirm the important role of FasL in NFATc1‐induced antiproliferative and pro‐apoptotic effects in HCC cells.

## 4 DISCUSSION

Over the past 20 years, the NFAT family has been shown to possess important functions, not only in immune cells but also in solid tissues and cells.^26^, ^27^ Mounting evidence suggests the NFAT family plays a key role in many types of tumorigenesis.^17^, ^18^, ^20^, ^28^, ^29^ In this study, we first determined expression of NFAT family members in HCC and found that NFATc1 mRNA exhibited the biggest difference in expression levels between HCC and corresponding adjacent nontumor tissues. Next, we focused on NFATc1 to examine NFATc1 expression in both HCC tissues and cell lines. NFATc1 expression was decreased in HCC compared to corresponding nontumor liver tissues and was also downregulated in HCC cell lines. These results suggest that NFATc1 silencing might be important for initiation and progression in HCC.

To better understand the role of NFATc1 in HCC, we analyzed the relationship between NFATc1 expression and clinical parameters. We found that low expression of NFATc1 in HCC was significantly associated with larger tumor size, advanced TNM stage, higher serum AFP levels, and liver cirrhosis. The relationship between low expression of NFATc1 and larger tumor size suggests that the decline in NFATc1 expression may help facilitate rapid tumor expansion. Additionally, decreased NFATc1 expression was correlated with advanced TNM stage, which may further promote HCC progression. In addition, our Kaplan‐Meier survival analysis revealed that low NFATc1 expression was significantly related to poor prognosis in patients with HCC. These results suggest that NFATc1 might play a tumor suppressor role in HCC tumorigenesis and progression and may be a good predictor of prognosis for patients with HCC after surgical resection.

As NFATc1 was downregulated in HCC, we investigated whether ectopic expression of NFATc1 could restore its tumor suppressor function and inhibit tumor growth. As Huh7 and PLC cell lines had the lowest NFATc1 expression, we transfected NFATc1 cDNA plasmid into these cells to explore its role in HCC. Ectopic expression of NFATc1 significantly suppressed both cell proliferation and colony formation. Subsequently, we observed that ectopic expression of NFATc1 in HCC cells led to G1 phase cell cycle arrest and induction of apoptosis. Together, these results indicate that NFATc1 might function as a tumor suppressor in HCC.

Some studies have demonstrated NFATc1 activation in malignant transformations, such as Burkitt lymphoma, melanoma, pancreatic, colorectal carcinomas, lung cancer, and breast cancer.^16^, ^17^, ^18^, ^29^, ^30^, ^31^ Wang et al^32^ also found that NFATc1 was overexpressed in HCC and promoted proliferation in HepG2 cell lines. However, other studies suggested NFATc1 might function as a tumor suppressor in some types of cancer.^33^, ^34^, ^35^, ^36^, ^37^, ^38^ Lucena et al^39^ found that different murine Nfatc1 isoforms played distinct roles in NIH 3T3 cells. While Nfatc1 isoform 3 induced cell transformation, Nfatc1 isoform 1 reduced cell proliferation and induce apoptosis. The distinct roles of NFATc1 in diverse cancer types might be explained by differential expression of NFATC1 isoforms in these tumors. Research from Wang et al reports different observations on NFATc1's role than our study, which might be the result of study on different NFATc1 isoforms. In our study, we found that NFATc1 (including NFATc1 isoforms B, C, D, E, F, and J) was downregulated in HCC, and TCGA data analysis revealed a similar trend. We then constructed a NFATc1 isoform B expression plasmid and transfected it into two HCC cell lines to explore NFATc1's function. Ectopic expression of NFATc1 isoform B indeed suppressed HCC cell proliferation while inducing apoptosis. Whether other NFATc1 isoforms have differential roles in HCC needs to be further investigated.

Furthermore, to understand how NFATc1 regulates downstream proteins to exert its tumor suppressor function, we performed qRT‐PCR analysis to examine candidate downstream genes that have important functions in tumor proliferation, apoptosis, and angiogenesis. We observed that ectopic expression of NFATc1 elevated MAT1A and FasL mRNA expression. We further demonstrated that NFATc1 induced FasL expression by directly binding to the FasL promoter and enhancing FasL promoter activity. Correlation analysis by IHC and TCGA dataset also revealed that there were positive correlations between NFATc1 and FasL. Consistent with our results, many studies have shown that NFATc1 binds FasL promoter to induce its expression in many other types of cells and tissues.^39^, ^40^, ^41^ Both dysregulation of the extrinsic and intrinsic apoptosis pathways are well known to be involved in cancer.^42^ FasL is a key protein that triggers the extrinsic apoptosis pathway,^43^ and its expression is regulated by many transcriptional factors such as Jun, NF‐κB, NFAT, EGR‐3, and SP1 in human tumors.^44^, ^45^, ^46^, ^47^, ^48^ Previous studies demonstrated that transfection of FasL into HepG2 cells induces apoptosis.^49^ The extrinsic and intrinsic apoptotic pathways are mediated by different caspases. Caspase 8 is the initiator in the extrinsic apoptotic pathway, while caspase 9 is the initiator in the intrinsic apoptotic pathway.^50^ We demonstrated that NFATc1 induced apoptosis through a caspase‐dependent pathway including caspase 8 activation, an initiator caspase of the extrinsic apoptotic pathway, followed by downstream executioner caspase 3 activation. However, caspase 9, an initiator caspase of the intrinsic apoptotic pathway, was not activated in NFATc1‐induced apoptosis. These data indicate that ectopic expression of NFATc1 in HCC cells leads to apoptosis by increasing FasL expression and activating the extrinsic apoptotic pathway.

Finally, in order to verify the important role of FasL in NFATc1's antiproliferative, pro‐apoptotic effect on HCC cells, we cotransfected an NFATc1 expression plasmid and a FasL siRNA in Huh7 and PLC cells. We found that knock‐down of FasL expression significantly impeded antiproliferative and pro‐apoptotic effects induced by NFATc1. These studies further confirm the important role of FasL in NFATc1's effects on HCC cells.

**Figure 7:**
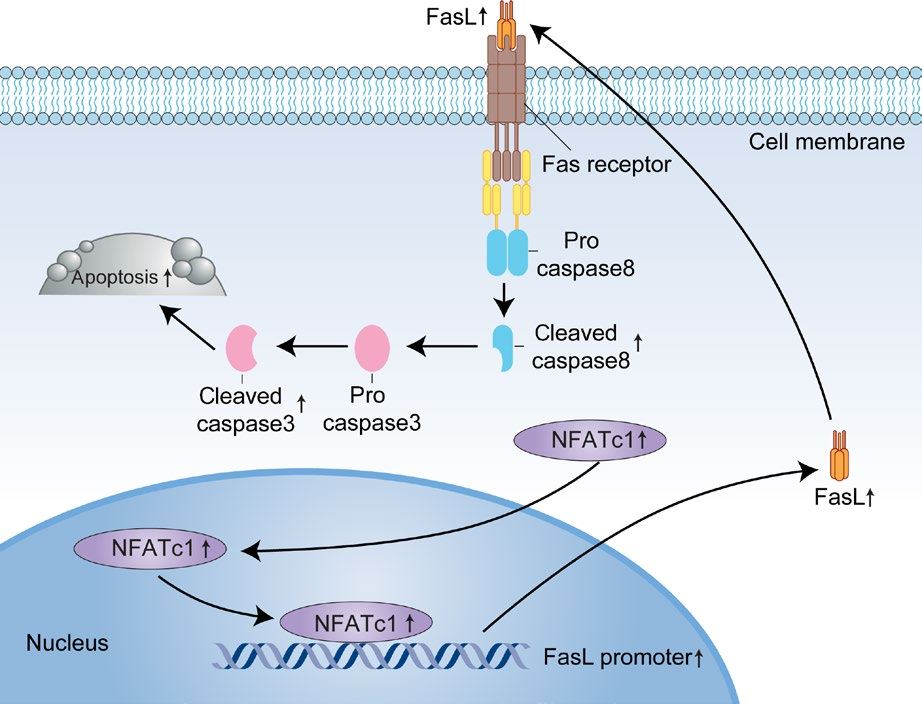
Schematic for NFATc1‐induced apoptosis in HCC cells through activation of the FasL‐mediated extrinsic signaling pathway. NFATc1 expression is silenced in HCC. Ectopic expression of NFATc1 induced FasL expression through direct binding to its promoter and induction of apoptosis signaling, including caspase 8 and caspase 3 activation, eventually leading to apoptosis

In summary, we revealed that NFATc1 is effectively silenced in HCC. Ectopic expression of NFATc1 inhibits cell growth and induces apoptosis. NFATc1 induced apoptosis by activating the FasL‐mediated extrinsic signaling pathway (Figure 7). Understanding the role of NFATc1 and its underlying mechanism in HCC might facilitate development of novel therapeutics for improving the prognosis of patients with HCC.

## CONFLICT OF INTEREST

The authors declare no conflict of interests.
