# Supplementary figures and images for: NFATc1 is a tumor suppressor in hepatocellular carcinoma and induces tumor cell apoptosis by activating the FasL‐mediated extrinsic signaling pathway
Source: Cancer Med. 2018 Aug 7;7(9):4701–17. doi: 10.1002/cam4.1716 (PMC6143940; doi:10.1002/cam4.1716)

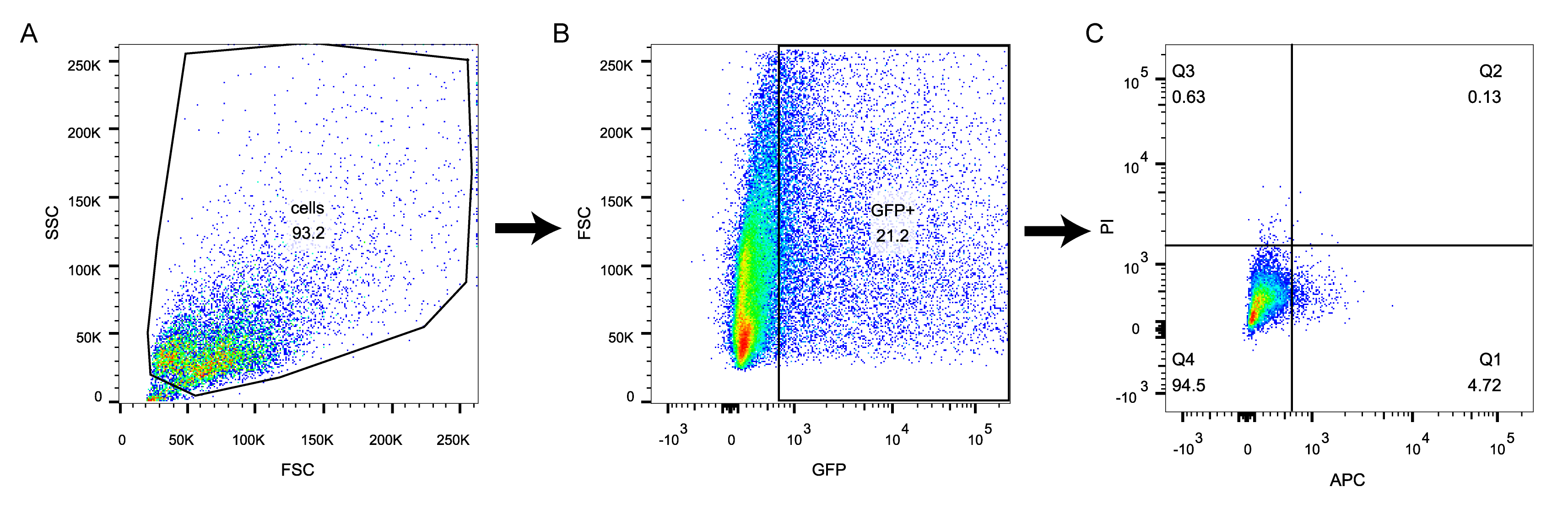

Supplement: Supplementary file 1 [file CAM4-7-4701-s001.tif]

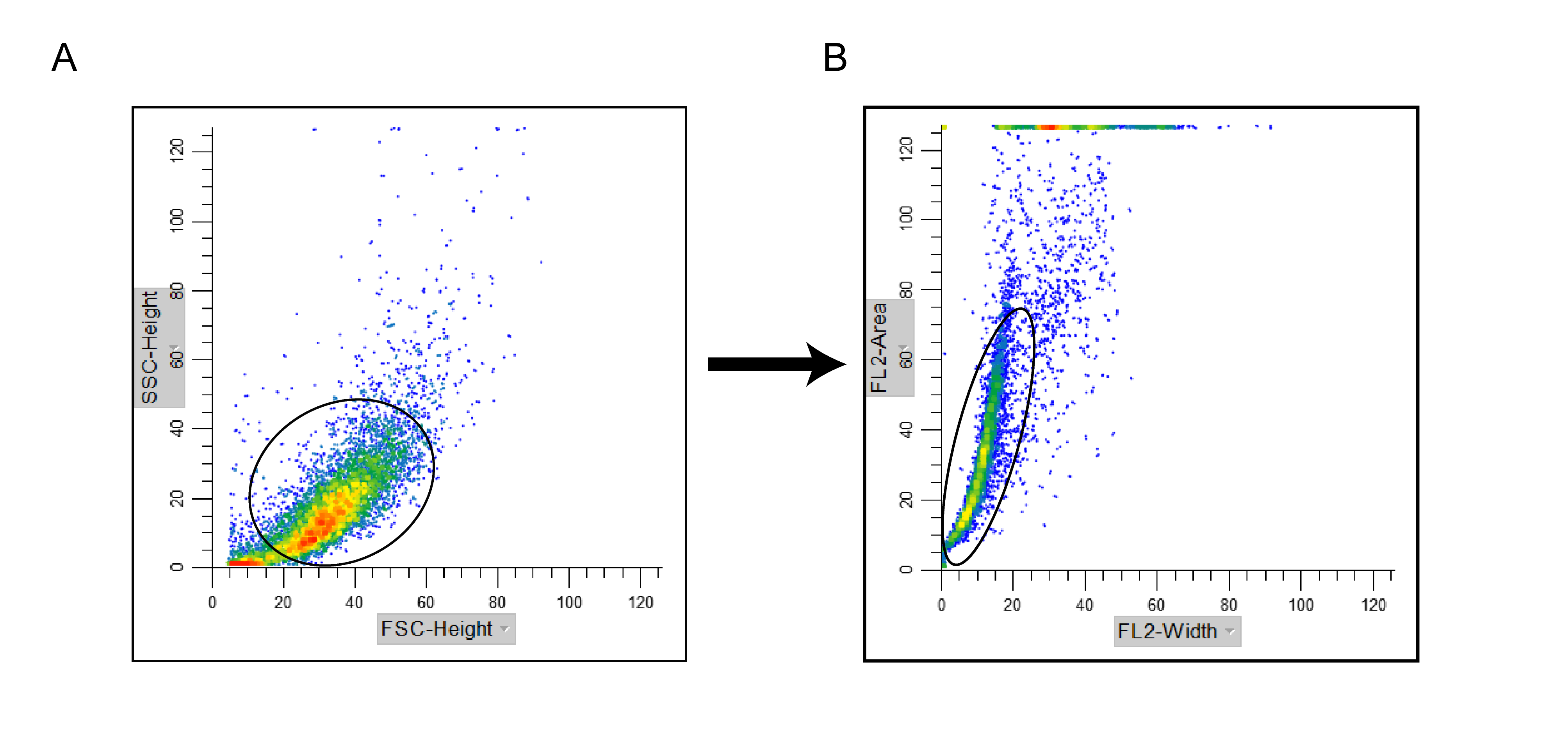

Supplement: Supplementary file 2 [file CAM4-7-4701-s002.tif]

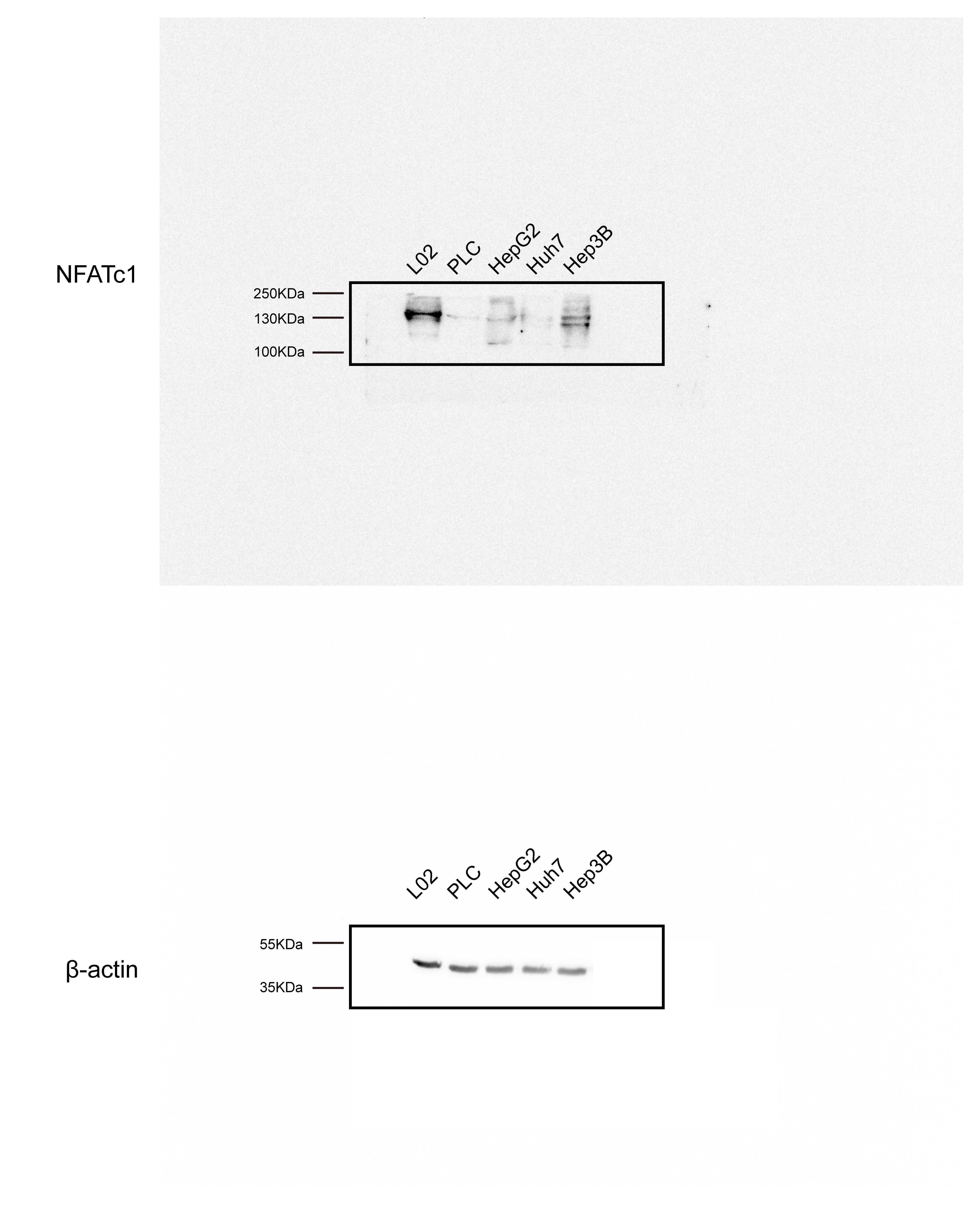

Supplement: Supplementary file 3 [file CAM4-7-4701-s003.tif]

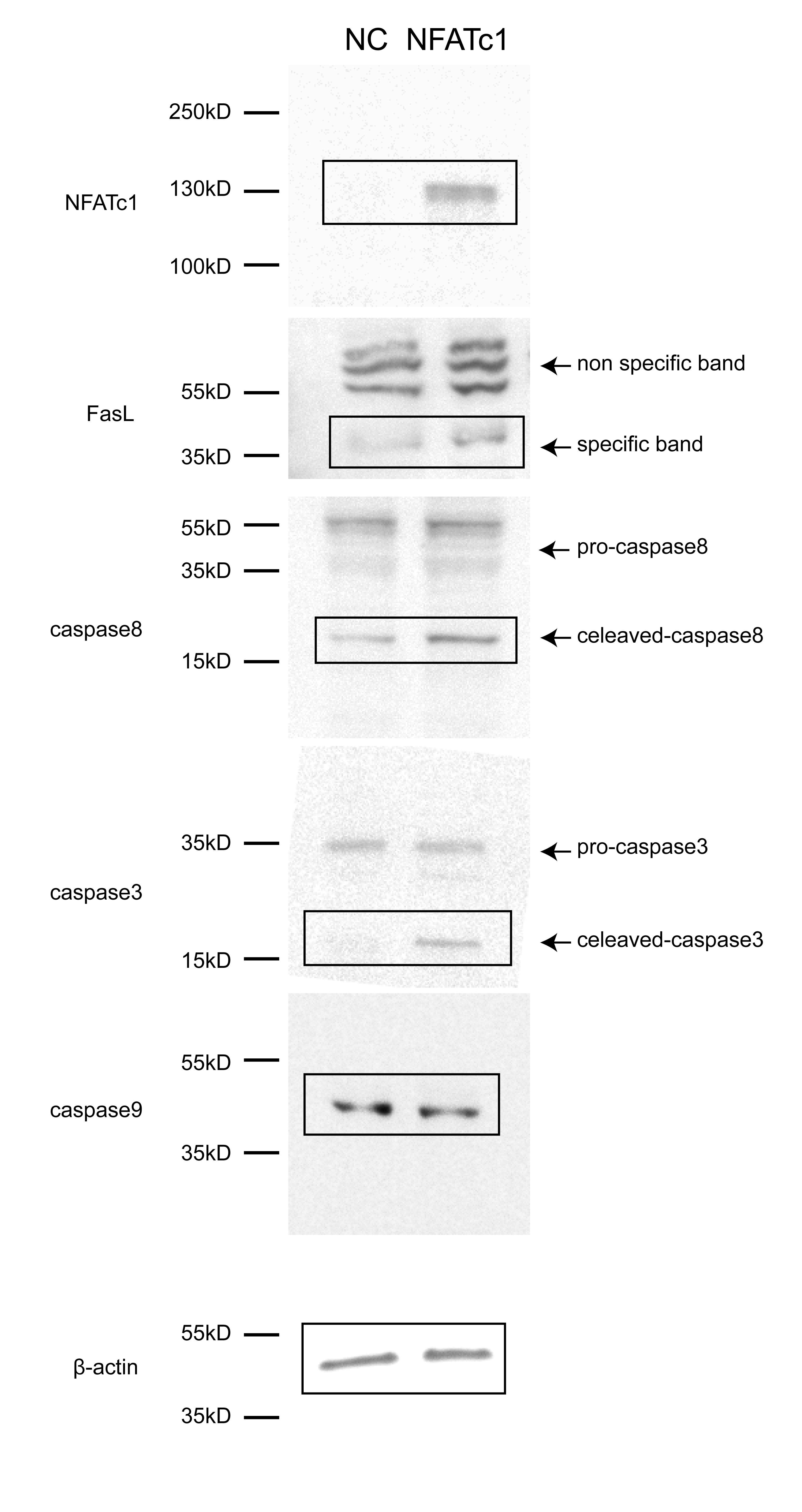

Supplement: Supplementary file 4 [file CAM4-7-4701-s004.tif]

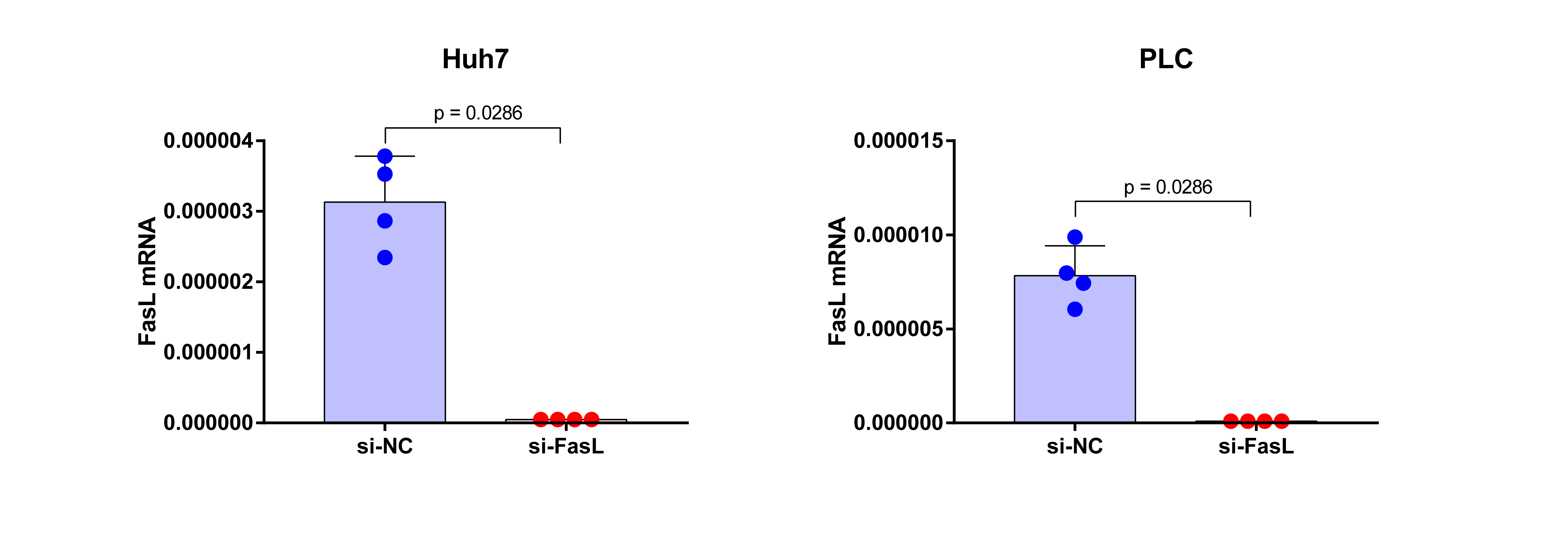

Supplement: Supplementary file 5 [file CAM4-7-4701-s005.tif]
