# Supplementary material for: NFATc1 is a tumor suppressor in hepatocellular carcinoma and induces tumor cell apoptosis by activating the FasL‐mediated extrinsic signaling pathway
Source: Cancer Med. 2018 Aug 7;7(9):4701–17. doi: 10.1002/cam4.1716 (PMC6143940; doi:10.1002/cam4.1716)
Supplement: Supplementary file 6 [file CAM4-7-4701-s006.docx]

Figure legend

**Supplementary Figure 1: Gate strategy of apoptosis analysis by flow cytometry.** (A) Cells expressing low FSC and SCC signal regarded as debris were excluded from further analysis. (B) Cells expressing positive GFP expression were regarded as successful transfected cells for further analysis. (C) quad gate set: Cells transfected with NFATc1 expression plasmid or Vector that not stained with APC or PI were considered as both APC and PI negative. We set quad gate for this rule.

**Supplementary Figure 2: Gate strategy of cell cycle analysis by flow cytometry.** (A) Cells expressing low FSC and SCC signal regarded as debris were excluded from further analysis. (B) Cells expressing high FL2 Width and FL2 Area regarded as Aggregated cells were excluded from further analysis.

**Supplementary Figure 3: Western Blot original figure presentation.** This figure is original figure of Figure 2E.

**Supplementary Figure 4: Western Blot original figure presentation.** This figure is original figure of Figure 4B. FasL had non-specific and specific band. Caspase-8 and Caspase-3 both had pro-caspase (caspase precursor) and cleaved-caspase band.

**Supplementary Figure 5: FasL siRNA knock-down efficiency.** FasL mRNA was measured by qRT-PCR one day after FasL siRNA was transfected into Huh7 cells. Si-NC was used as a negative control. Statistical analysis was performed using the Mann–Whitney U test. Data are presented as the mean ± SD.
